# 2698. Trends of Antibiotic Resistance in *E. coli, Klebsiella Spp., Pseudomonas aeruginosa and Enterococcus spp.* Urinary Tract Infection in Renal Transplant Recipients from Pakistan over a Decade

**DOI:** 10.1093/ofid/ofad500.2309

**Published:** 2023-11-27

**Authors:** Sunil Kumar Dodani, Asma Nasim

**Affiliations:** Sindh Institute of Urology and Transplantation, Karachi, Sindh, Pakistan; Sindh Institute of Urology and Transplantation, Karachi, Sindh, Pakistan

## Abstract

**Background:**

Antimicrobial resistance is a major public threat worldwide. In Pakistan many studies reported a rising trend of resistance to antimicrobials in clinical isolates of *Klebsiella species (spp.)*, *E-coli*, *Pseudomonas aeruginosa and Enterococcus spp.* Solid organ transplant (SOT) recipients are more prone to infections, particularly with MDR organisms due to immunocompromised status, multiple hospital visits and increase exposure to antibiotics. There is paucity of data on antibiotic susceptibility pattern among renal transplant recipients from Pakistan. This study is conducted to find the trends in antimicrobial resistance pattern among common organisms isolated in urinary tract over a 10-year period in renal transplant recipients. The aim is to guide the physicians regarding decision over empirical antimicrobial choices.

**Methods:**

A retrospective computerized data review of urine cultures from renal transplant recipients in 2010 and 2020 was conducted. The trend and sensitivity patterns of *E.coli, Klebsiella spp. Pseudomonas aeruginosa and Enterococcus spp* was collected. The resistance pattern of diffferent antibiotics were compared over the 10 years period.

**Results:**

A total of 2,088 out of 6,249 (33%) and 2902 out of 8,115 (36%) urine cultures were positive in 2010 and 2020 respectively. The most common organisms isolated and their trend in the last decade is shown in figure 1. Over the decade, E-coli strains become 100% resistant to ciprofloxacin, ceftriaxone resistance has increased to 85% and imipenem to 10%. In *Klebsiella spp*. imipenem resistance has increased to 14%. There is no significant change in resistance pattern among *Pseudomonas aeruginosa.* Regarding *Enterococcus spp*. there is a rise in vancomycin resistance over the decade from 13% to 17%. (fig. 2)

Trends of organism over decade

**Figure:**
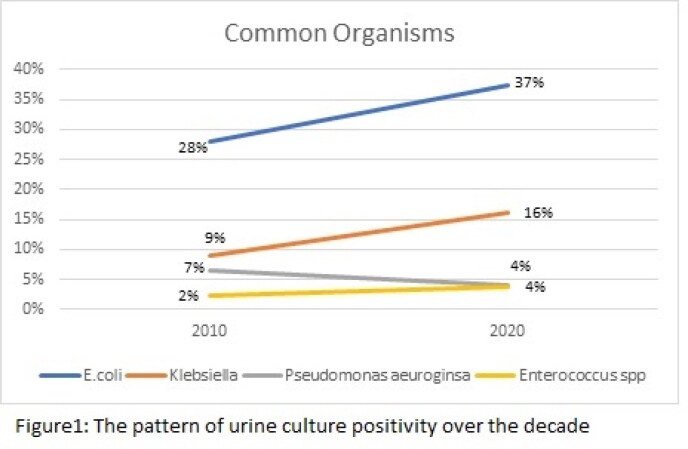

**Figure:**
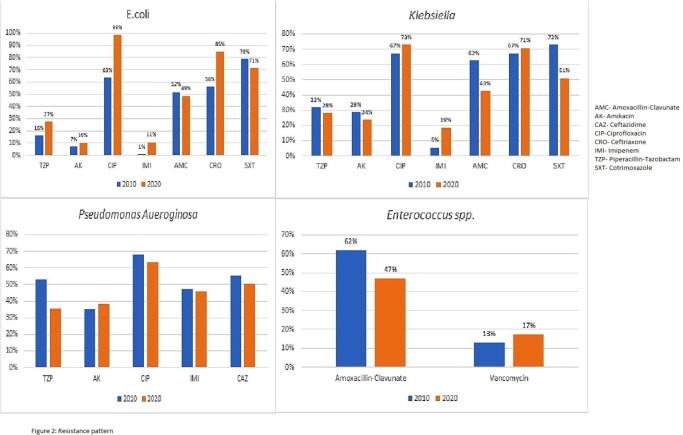

**Conclusion:**

Resistance to broad spectrum antimicrobials has been increased with >10% increase in carbapenems resistance over the last 10 years. E-coli have particularly become more resistant over the decade. We are also seeing more vancomycin resistant enterococcus. The alarming increase in resistance may lead to increased morbidity in renal transplant recipients because we left with only injectable options. We need a robust stewardship program for judicious use of antimicrobials to contain increasing resistance.

**Disclosures:**

**All Authors**: No reported disclosures

